# Health care professionals’ understanding of contraindications for physical activity advice in the setting of stem cell transplantation

**DOI:** 10.1007/s00520-022-07336-4

**Published:** 2022-08-29

**Authors:** Corinna Meyer-Schwickerath, Maximilian Köppel, Rea Kühl, Janina Bujan Rivera, Angeliki Tsiouris, Gerhard Huber, Joachim Wiskemann

**Affiliations:** 1 Institute of Sports and Sports Sciences, University of Heidelberg, Heidelberg, Germany; 2 Working Group Exercise Oncology, Department of Medical Oncology, National Center for Tumor Diseases (NCT), Im Neuenheimer Feld 460, 69120 Heidelberg, Germany; 3 Department of Psychosomatic Medicine and Psychotherapy, University Medical Center Mainz, Mainz, Germany

**Keywords:** Physical activity counseling, Contraindications, Hematopoietic stem cell transplantation, Physical activity, Exercise, Health care professionals

## Abstract

**Purpose:**

Most patients receiving a hematopoietic stem cell transplantation (HSCT) are able to tolerate and benefit from physical activity (PA). Therefore, it is important that health care professionals (HCPs) advise patients to perform PA before, during, and after transplantation. By understanding which medical conditions and safety issues are associated with the (non-) promotion of PA, concrete actions and interventions can be planned and implemented.

**Methods:**

Physicians (*N* = 51), nurses (*N* = 52), and physical therapists (*N* = 26) participated in a nationwide cross-sectional online survey. HCPs’ understanding of 15 medical conditions as contraindications for PA was assessed. Significant group differences were determined using chi-square analysis.

**Results:**

*Acute infection* was the only condition which was considered as contraindication by all HCPs (62.7%). C*achexia* (78%), *having a stoma* (91%), *or port* (96.2%), *psychological problems* (88.4%), and *leukopenia* (83.3%) were not considered as contraindications. Six conditions were rated inconsistently between the groups, whereas physicians had the least concerns regarding PA. Physicians with an additional training in PA perceived a *platelet count of* ≤ *50,000/μl* significantly less often as contraindication (*p* < 0.05).

**Conclusion:**

The large number of potentially-answers especially in nursing staff and physical therapists might reflect caution or uncertainty. There is a clear need for a good multidisciplinary cooperation between all HCPs in order to support patients to confidently engage in PA. Furthermore, education possibilities and evidence-based courses to build knowledge regarding safety concerns should be the standard practice in the setting of HSCT. The investigative nature of the paper indicates that certain trends should be interrogated in a causal-longitudinal design.

## Introduction

Hematopoietic stem cell transplantation (HSCT) presents an important treatment option for many hematological malignancies [1]. As treatment procedures and measures improve, the use of HSCT continues to increase worldwide with about 50,000 people receiving an HSCT each year. The combination of a concomitant rise in the number of survivors and high treatment-related burden due to numerous side effects highlights the growing need for evidence-based adjuvant therapy options [2]. Physical activity has been shown to positively affect physical and psychosocial function and QoL in HSCT patients [3]. Furthermore, programs offered during treatment seek to minimize treatment-related side effects and potentially impact patients’ prognosis [4–6]. Since physical deconditioning is highly prevalent in HSCT patients [7], promotion of health-enhancing physical activity (PA) should be one of the key components of supportive care [8].

Literature suggests that cancer survivors tend to be more physically active if they receive an exercise recommendation, or PA counseling [9, 10]. Thus, health care professionals (HCPs) should encourage survivors to perform PA before, during, and after transplantation [5]. However, discussing PA with cancer survivors is not yet the standard practice within oncology care [11].

Different types of barriers which might prevent HCPs from promoting PA are described in previous studies [12, 13]. Besides structural barriers, such as lack of times or missing guidelines, safety issues can hinder HCPs to recommend PA. Current data suggests that there are only few absolute contraindications to exercise testing and training, as for example instable angina or acute infections [14]. Most patients with cancer will only exhibit relative contraindications [15]. Furthermore, guidelines for cancer survivors’ exercise prescription provided by the American College of Sports Medicine did not report any general contraindications to starting a PA program for patients undergoing HSCT [16]. This means that the prescription, the setting, and supervision has to be appropriate and individually adjusted [15].

By understanding which medical conditions and safety issues are associated with the (non-) promotion of PA among physicians, nursing staff, and physical therapists, concrete actions and interventions can be planned in order to positively influence these factors. A profound understanding of the attitudes and uncertainties of HCPs toward medical conditions as contraindications might help to detect where the greatest need for further research and education lies.

To our knowledge, the setting of HSCT has not been investigated regarding HCPs’ understanding of contraindications for PA advice. Consequently, we conducted a nationwide survey in Germany (1) to investigate to what extent specific medical conditions and safety issues are perceived as contraindications to PA in the setting of HSCT, and (2) to explore whether HCPs differ in their understanding. Additionally, we examined the role of sociodemographic factors and participants’ characteristics on HCPs’ understanding of medical contraindications for PA advice.

## Methods

### Study design

This cross-sectional study, an anonymized online survey, was conducted between November 2020 and October 2021. The results presented in this manuscript were part of the COHRACT project, a nationwide online survey of HCPs in German stem cell transplantation centers. The study focused on the attitudes and counseling behavior of physicians, nurses, and physical therapists regarding PA in the setting of HSCT. The study protocol was approved by the Medical Ethic Committee of the University of Heidelberg (reference number: S-150/2018). The questionnaire was inspired by and is partly consistent with the cross-sectional surveys used in the MOMENTUM Project assessing influential medical and psychosocial factors for PA among HCPs and persons with cancer [17–19].

### Participants

Physicians, nurses, and physical therapists treating and caring for patients receiving an HSCT were asked to participate. Participants were eligible if they were ≥ 18 years old. The present analysis was restricted to individuals who provided information concerning their understanding of specific medical conditions and safety issues. Participants anonymously completed the online survey after providing informed consent.

### Participant recruitment

Different recruiting strategies were applied. For initial recruitment of HCPs, the group-specific questionnaires were distributed using the mailing list of the working group “Kooperative Transplantationsstudiengruppe” of the DAG KBT (Cooperative transplant study group) and the mailing list of the German-speaking myeloma multicenter group (GMMG). Additionally, the first author contacted all German stem cell transplantation centers (*N* = 58) by phone. Responsible contact persons were identified and provided with study information via email. Four to 6 weeks later a reminder email was sent.

### Measures

Information was self-reported by HCPs in group-specific online questionnaires. Participants were presented the following list of 15 medical conditions/safety issues: acute infection, osteolysis, increasing pain occurring during PA, platelet count of ≤ 50,000/μl, port, cachexia, leukopenia, no prior physical capability test, hemoglobin < 8 g/dl, stoma, graft versus host disease grade ≥ II (GvHD), steroid/glucocorticoids administration, cardiac insufficiency, pulmonary disease, and psychological problems. For every condition, participants were asked: “Does this represent a medical contraindication for PA during or after HSCT?” Response options were no, potentially, or yes. The scale is partly consistent with the scale used in the cross-sectional survey of the MOMENTUM Project [17–19]. The scale was qualitatively and quantitatively pretested before using it in the MOMENTUM Project [20] and additionally pretested in a qualitative pilot study in the specific setting of HSCT (physicians *N* = 11, nursing staff *N* = 7, physical therapists *N* = 2). The present study focuses exclusively on quantitative elements of the survey. The selection of the particular medical conditions was primarily based on safety issues described in the ACSM roundtable on exercise guidelines for cancer survivors [16]. Additionally, medical conditions stated in the National Comprehensive Cancer Network guidelines [21] were considered as well as frequently mentioned exclusion criteria in exercise and PA intervention studies with patients receiving HSCT.

### Data analysis

Descriptive analysis was used to determine demographic and medical characteristics and HCPs’ understanding of contraindications. Continuously measured characteristics were split into categorical variables.

Significant group differences between HCPs in their understanding of medical conditions were determined using chi-square tests. Strength of association in this comparison was evaluated using contingency coefficient (*C*). Contingency coefficients (*C*) of 0.10, 0.30, and 0.50 represent small, medium, and large effects [22]. On the basis of calculated effect sizes, medical conditions were categorized in (1) judged with agreement, (2) judged with low disagreement, or (3) judged with medium disagreement across the HCP groups. Finally, if the chi-square tests revealed significant differences between the three groups, pairwise comparisons were conducted using Fisher’s exact test.

In order to discover whether an additional professional training in the field of PA influences the understanding of specific possible contraindications for PA, further group comparisons were conducted by dividing HCPs into two groups: HCPs with and without additional PA training. Group differences were calculated using chi-square tests and Cramer’s V (V) as indicator for strength of association. Effect sizes of 0.10, 0.30, and 0.50 were considered as small, medium, and large effects [22]. For comparing individuals in their overall understanding of medical contraindications, an individual aggregated value was calculated. Therefore, all ratings given by one participant were put in ratio by weighting the number of no-, potentially-, and yes-answers with a factor [(sum no-answers*0 + sum potentially answers*0.5 + sum yes-answers*1)/number of valid answers]. By means of this aggregated contraindication score, subgroups were compared in their overall tendency to rate the influence of medical conditions rather permissively or rather strictly, using analyses of variance and *t*-tests for independent samples. A post hoc test (Tukey–Kramer test) was used to determine which pairwise comparisons are significant. Statistical analyses were carried out with IBM SPSS version 24.

## Results

### Recruitment flow

**Fig. 1:**
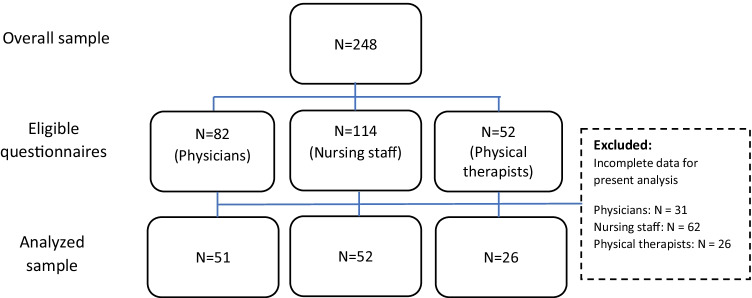
Recruitment flow. Abbreviations: N, number

Recruitment flow is shown in Fig. 1. In total, we received 248 eligible questionnaires from HCPs. After removal of cases due to incomplete data for the present analysis, a total of 129 surveys were analyzed.

### Descriptive characteristics

Descriptive characteristics of the samples are displayed in Table 1 providing sample characteristics of physicians (*N* = 51), nursing staff (*N* = 52), and physical therapists (*N* = 26).

**Table 1:** Descriptive statistics of the HCPs’ sample characteristics (*N* = 129)

Characteristics	*M* or *N*			SD or %		
	Physicians (*N* = 51)	Nurses (*N* = 52)	Physical therapists (*N* = 26)	Physicians	Nurses	Physical therapists
Sex						
Female	25	41	19	49%	78.8%	73.1%
Male	26	11	7	51%	21.2%	26.9%
Age	43.7	40.7	43.2	10.61	12.1	13.2
Age categories						
Until 30	6	12	7	11.8%	23.1%	26.9%
Until 50	30	29	6	58.8%	55.8%	23.1%
Until 70	15	11	13	39.4%	21.2%	50%
BMI	22.97	27.6	22.4	2.7	5.98	2.4
BMI categories						
Less than 18.5 (underweight)	3	1		5.9%	2%	
18.5–24.9 (normal weight)	39	19	22	76.50%	37.3%	84.6%
25–29.9 (overweight)	9	15	4	17.6%	29.4%	15.4%
30 + (obese)		16			31.4%	
Work experience (years)	17.0	16.88	20.96	10.31	11.65	12.9
< 10 years	17	12	8	33.3%	23.1%	30.8%
< 20 years	17	8	3	33.3%	15.4%	11.5%
< 30 years	11	10	8	21.6%	19.2%	30.8%
30 +	6	22	7	11.8%	42.3%	26.9%
Position*						
Assistant doctor/in training	12	36	23	23.5%	76.6%	88.5%
Specialist physician/nurse	8	11	3	15.7%	23.4%	11.5%
Senior physician	26			51.0%		
Chief physician/head nurse	5			9.8%		
Professional environment						
University hospital	48	49	24	94.1%	94.2%	92.3%
Other hospital	3	3	2	5.9%	5.8%	7.7%
Additional PA training^a^						
Yes	9	13	20	17.6%	25.0%	76.9%
No	42	39	6	82.4%	75.0%	23.1%
Working focus						
Allogenic	32			64%		
Autologous	18			36%		
Moderately active within past 4 weeks						
Yes	46	38	24	90.2%	74.5%	92.3%
No	5	13	2	9.8%	25.5%	7.7%
Aerobic exercise minutes/week	270.5	342.6	307.5	164	270	186.6

^a^HCPs were asked if they had participated in any continuing education or training in physical activity

Abbreviations: *M* mean, *N* number, *SD* standard deviation, *BMI* body mass index

^*^Choices for physical therapists: in training, physical therapist, leadership position

### Similarities and differences in the understanding of medical conditions between HCPs

Table 2 displays descriptive results and presents group differences between HCPs. For nine conditions, there were no significant differences, and thus a general agreement between the subgroups. Precisely, there was a general agreement that cachexia (78%), having a stoma (91%), or port (96.2%), psychological problems (88.4%), or leukopenia (83.3%) do not represent a contraindication to PA. For having a platelet count below 50,000/μl (56.8%) or GvHD ≥ II (62.6%), most participants agree that they do not represent a contraindication to PA. However, the proportion of participants choosing “partially” was also quite high (with up to 36%). Increasing pain occurring during PA turned out to be perceived most cautiously, as measured by the high proportion of potentially-answers (64.6%). Acute infection was rated as contraindication by most participants (62.7%).

**Table 2:** Frequencies (in percent) of HCPs on medical conditions

“Do the below defined medical conditions represent a contraindication for physical activity?”	Physicians			Nursing staff			Physical therapists			Statistical test of group differences			
	*N* = 52			*N* = 52			*N* = 26						
	No (%)	Pot (%)	Yes (%)	No (%)	Pot (%)	Yes (%)	No (%)	Pot (%)	Yes (%)	*χ*^2^	*p*-value	Effect size (*C*)^a^	Sign. group diff. between^b^
Agreement between groups and certainty in how to rate medical condition													
Platelet count ≤ 50,000/μl	62.7	33.3	3.9	50.0	42.3	7.7	57.7	30.8	11.5	2.06	0.72	0.14	-
Cachexia	80.4	19.6	0	75.0	21.2	3.8	80.8	19.2	0	3.15	0.53	0.17	-
Stoma	98.0	2.0	0	90.4	5.8	3.8	84.6	7.7	7.7	3.74	0.44	0.18	-
GvHD ≥ II	72.5	27.5	0	57.7	38.5	3.8	57.7	42.3	0	4.63	0.33	0.20	-
Port	100	0	0	96.2	0	3.8	92.3	3.8	3.8	4.91	0.3	0.21	-
Acute infection	2	49	49	0	23.1	76.9	3.8	30.8	65.4	5.52	0.24	0.22	-
Psychological problems	86.3	13.7	0	90.4	5.8	3.8	88.5	11.5	0	5.62	0.23	0.22	-
Increasing pain occurring during PA	5.9	78.4	15.7	15.4	50.0	34.6	15.4	65.4	19.2	8.24	0.08	0.27	-
Leukopenia	96.1	3.9	0	80.8	15.4	3.8	73.1	23.1	3.8	8.34	0.08	0.27	-
Low disagreement between groups in how to rate medical condition													
Cardiac insufficiency	64.7	33.3	2.0	26.8	65.4	7.7	46.2	53.8	0	10.28	0.036	0.29	P < N
No prior physical capability test	94.1	3.9	2.0	76.9	19.2	3.8	53.8	34.6	11.5	12.67	0.013	0.32	P < N, P < Phy
Osteolysis	25.5	70.6	3.9	5.8	65.4	28.8	30.8	65.4	3.8	15.56	0.004	0.36	P < N, N > Phy
Pulmonary disease	66.7	31.4	2.0	25.0	65.4	9.6	53.8	46.2	0	17.15	0.002	0.37	P < N, N > Phy
Hemoglobin < 8 g/dl	60.8	37.3	2.0	32.7	63.5	3.8	19.2	57.7	23.1	19.68	0.001	0.39	P < N, P < Phy, N < Phy
Steroid/glucocorticoids administration	98.0	2.0	0	86.5	9.6	3.8	61.5	34.6	3.8	24.89	0.000	0.43	P < Phy, N < Phy

^a^Contingency coefficients (*C*) of 0.10, 0.30, and 0.50 represent small, medium, and large degrees of association

^b^Based on Fisher’s exact test. All group differences (*N* = 12) are significant at *p* < 0.05; *N* = 6 group differences are significant at *p* < 0.001

Abbreviations: *Pot* potentially, *sign* significant, *GvHD* graft versus host disease, *P* physicians, *N* nursing staff, *Phy* physical therapists

< 0.001 cardiac insufficiency P-N, pulmonary disease P-N, steroid/glucocorticoids administration P-Phy, hemoglobin P-Phy, osteolysis P-N, no prior physical capability test P-Phy

< 0.05 osteolysis N-P, no prior physical capability test P-N, hemoglobin P-N, hemoglobin N-Phy, steroid/glucocorticoids administration N-Phy, pulmonary disease N-Phy

The remaining six medical conditions revealed significant differences in HCPs’ understanding and display low disagreement between groups. Nursing staff tended to have a higher number of potentially-answers and thus evaluated more medical conditions with a cautious approach than physicians did (cardiac insufficiency, no prior physical capability test, osteolysis, pulmonary disease, hemoglobin < 8 g/dl). In three medical conditions, physical therapists had a significantly higher number of potentially-answers in comparison to physicians (no prior physical capability test, hemoglobin < 8 g/dl, and steroid/glucocorticoids administration). Furthermore, nursing staff rated “osteolysis” significantly more often as contraindication and “pulmonary disease” significantly more cautiously than physical therapists, whereas physical therapists rated “hemoglobin < 8 g/dl” significantly more often as contraindication, and “steroid/glucocorticoids administration” with greater concerns in comparison to nursing staff.

### Comparison of understanding of medical conditions among HCPs with and without PA training

**Fig. 2:**
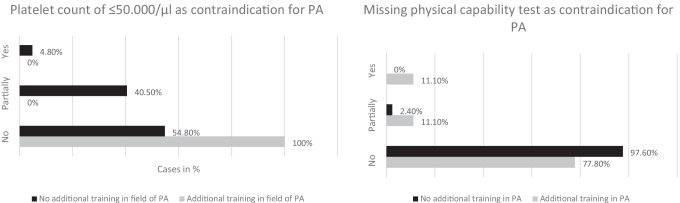
Comparison of physicians’ response frequencies (in percent) on particular medical conditions with the grouping factor participants received additional professional training in the field of PA versus no such training. Differences are significant at *p* < 0.05

Significant group differences between physicians with or without an additional training in the field of PA are displayed in Fig. 2. Physicians with an additional training were more likely to not perceive a platelet count of less than 50,000/μl as a contraindication, whereas physicians without an additional training significantly less often considered “no prior physical capability test” as contraindication for PA.

### Differences in aggregated contraindication scores

Aggregated contraindication scores (mean and standard deviations) are displayed in Table 3. Additionally, differences in the overall understanding of medical conditions based on further characteristics of HCPs are displayed. Differences in understanding based on sex and age were calculated separately for all HCPs. Within these groups, there are no significant differences between male and female participants and the different age categories. HCPs differed significantly in their overall understanding of medical conditions, depending on their professional background [*F* (2,126) = 10.92, *p* < 0.001, *η*2 = 0.15]. Physicians had the lowest, while nurses had the highest aggregated contraindication score (*M* = 0.19 vs. 0.30).

**Table 3:** Differences in aggregated contraindication scores (M/SD) based on demographic and professional variables

	M (SD)	F	*t*	*p*-value	Effect size^b^
Physicians					
Sex					
Male (*N* = 25)	0.20 (0.10)		− 1	0.229	0.341
Female (*N* = 26)	0.17 (0.08)				
Age					
< 45 years (*N = *29)	0.19 (0.08)		0	0.730	0.097
> 45 years (N = 22)	0.19 (0.10)				
Nursing staff					
Sex					
Male (*N* = 11)	0.29 (0.11)		0.071	0.944	0
Female (*N* = 41)	0.30 (0.16)				
Age					
< 45 years (*N* = 35)	0.32 (0.16)		1	0.070	− 1
> 45 years (*N *= 17)	0.24 (0.10)				
Physical therapists					
Sex					
Male (*N* = 7)	0.28 (0.19)		0	0.943	0.032
Female (*N* = 19)	0.28 (0.12)				
Age					
< 45 years (*N* = 12)	0.27 (0.09)		0	0.813	0.094
> 45 years (*N* = 14)	0.28 (0.14)				
Profession*		10.92		< 0.001	0.15
Physicians	0.19 (0.09)				
Nursing staff	0.30 (0.15)				
Physical therapists	0.28 (0.16)				

^b^Effect sizes: partial *η*2 for the ANOVA, *d* for *t*-tests

^*^Post hoc test (Tukey–Kramer test) revealed significant difference in aggregated contraindication score between P-N and P-Phy

## Discussion

To the best of our knowledge, this was the first cross-sectional study providing detailed insights into HCPs’ understanding of specific medical conditions and safety issues as contraindications to PA in the setting of HSCT. There was general agreement between HCPs that cachexia, having a stoma, or a port, psychological problems, and leukopenia do not represent contraindications to PA. Although answered with a more cautious approach, all groups agreed that having a platelet count of ≤ 50,000/μl and GvHD ≥ II do not represent a contraindication to PA. “Increasing pain occurring during PA” was rated most cautiously with a high proportion of potentially-answers by all participants. Remarkably, “acute infection” was the only condition which was predominantly considered as contraindication by all HCPs.

Physicians mostly had less concerns with respect to medical conditions being potential contraindications for PA. The discrepancy mainly results from the fact that nursing staff and physical therapists had higher numbers of potentially-answers. Interestingly, an additional training in the field of PA seems to influence physicians’ response behavior. Physicians with such additional training reported significantly more frequently that a “platelet count of ≤ 50,000/μl” does not represent a contraindication to PA and considered “prior physical capability tests” more often as necessary.

Historically, many medical conditions have been a concern and considered a contraindication for exercise and PA [23]. As previous studies have shown that most HSCT patients are able to tolerate and benefit from PA [3, 23], it is important that HCPs advise patients to perform PA before, during, and after transplantation [5]. Our data suggest that results of recent studies have been recognized by HCPs working in the field of HSCT, as only one medical condition (infections) was consistently considered as contraindication to PA. However, the large number of potentially-answers might reflect caution or uncertainty, and consequently influence HCPs’ PA counseling behavior. Unfortunately, there are still many gaps in the literature regarding safety issues [24]. There is very limited structured, empirical evidence, which clarifies the relevance of specific medical contraindications for PA [25]. Additionally, studies with strict exclusion criteria will scarcely contribute to increase knowledge about medical contraindications for PA among HSCT patients. Therefore, complications due to bed rest vs. benefits of PA have to be considered individually within the patient’s overall clinical picture.

Exercise guidelines for cancer survivors suggest pre-exercise medical evaluation and clearance by physicians in case of lung or abdominal surgery, ostomy, cardiopulmonary disease, ataxia, extreme fatigue, severe nutritional deficiencies, worsening/changing physical condition, and bone metastases [23]. Accordingly, assessing each patient through the lens of providing safe rehabilitation interventions in a medically supervised setting is required [24].

Although a significant difference was found between physicians with and without additional professional training or education in PA, the absolute majority does not consider a “missing physical capability test” as a contraindication for PA. The significant result is based only on individual voices within a small sample (*n* = 9). Nonetheless, the trend identified here may make it possible to speculate that education possibilities and courses to build knowledge regarding PA influences physicians’ understanding.

Only by incorporating assessment of basic functional ability as a routine component of HSCT care, HCPs can provide safe and effective PA counseling [26, 27].

Acute infection was the most commonly cited contraindication. As studies on animals show that strenuous exercise during an ongoing febrile infection can cause complications and increased lethality [28], it is absolutely reasonable that our data suggests a great agreement that PA during an infectious illness may lead to serious complications. Furthermore, consensus guidelines exist that rest from strenuous exercise is recommended for all individuals with a fever (≥ 38 °C) [29]. However, limited data report over the feasibility of individual incorporated supervised low-intensity PA regimens, all tailored to individual needs. For instance, Hedin et al. stated that patients performing a moderate PA program (getting out of bed every half hour during waking hours) during a febrile course did not experience the orthostatic declines or blood volume reductions that are normally induced by illness and bed rest without PA [30].

Interestingly, when comparing physicians’ response frequencies regarding an often-discussed safety issue like “platelet count of ≤ 50,000/μl,” physicians with an additional training in the field of PA agreed that this specific threshold does not represent a contraindication and were more permissive in their judgment. In former studies, varying platelet counts between < 20,000 and 50,000/μl have been considered as contraindication for exercise and participants were consequently disqualified from participation [31, 32]. Excluding these patients is mainly explained with thrombocytopenia potentially leading to hemorrhages [33]. However, recent studies have shown that most HSCT patients are able to tolerate and benefit from PA despite low platelet counts (< 10,000/μl) [34, 35]. A retrospective study by Fu et al. adapted the inpatient rehabilitation protocol with regard to platelet counts and designed therapy sessions addressing each patient’s individual impairments and rehabilitation goals [36]. The protocol implies, for example, no resistive exercise in patients with platelet counts of 10,000–20,000/μl. Inpatients with high fall risk standing or ambulating should be avoided. Inpatients with platelet counts of 5000–10,000/μl resistive exercises should be avoided and exercises should be limited to in bed or chair. They showed that the risk of severe exercise-related bleeding events was low and that potential risks must be assessed in conjunction with the negative effects of immobility and bed rest. Although reliable data regarding risk factors and feasibility during cytopenia are limited [37], preliminary findings should support HCPs to take the entire clinical situation of the patient into account, rather than solely relying on individual blood values [38].

Remarkably, not more than 4% of physicians and physical therapists considered the presence of osteolysis as contraindication. Although participating physicians and physical therapists seem to be aware of the potential and benefits of PA, the high proportion of “potentially-answers” might reflect uncertainties around safety and the overall risk of skeletal-related events. At the present time, fundamental limitations remain due to a lack of high-quality research on standardized approaches to predict the risk of skeletal complications (especially for people that might be at increased risk of complications as, e.g., in elderly individuals with multiple myeloma) [39]. However, just recently, a published expert recommendation statement paper for people with bone metastases, which might serve as initial orientation, concludes that even people with bone metastases should be supported and encouraged to engage in regular PA [39]. Weller et al. conclude in their systematic review that exercise appears safe and feasible for individuals with bone metastases when it includes an element of supervised instruction [40]. Until the development of specific PA guidelines for individuals living with bone metastases, and the provision of further information on the feasibility and safety of PA in patients with osteolysis, the perceived risk of skeletal complications should be weighed against potential health benefits [39].

Previous studies showed that safety issues can hinder physicians [41, 42] and nurses [43] to recommend PA to cancer patients. However, the majority of studies used single items in order to evaluate safety concerns [44] and only assessed whether participants considered exercise as a safe intervention during cancer treatment. Therefore, the results do not allow a direct comparison with our data. It is important to mention that in a cross-sectional study by Tsiouris et al. assessing HCPs’ perception of contraindications for PA during cancer treatment, the medical condition “acute infection” was also the only condition assessed as a clear contraindication by all groups. However, the study suggests even a higher level of cautiousness in judging particular medical conditions as contraindications for PA [20]. In our sample, there were three times as many conditions with agreement across groups and a clearer positioning of HCPs whether or not they considered a certain condition as contraindication to PA. In addition to our smaller sample size, a non-response bias might have influenced the response pattern.

Our study has several limitations. Recruiting participants was extremely difficult (presumably due to the higher workload during the COVID-19 pandemic). Therefore, a non-response bias has to be considered, as participants might have been especially aware of the importance of PA in the treatment of patients receiving HSCT (this assumption is also supported by including very physically active participants themselves). Furthermore, it is questionable to assess HCPs’ understanding of single medical conditions, as most HSCT patients present with a combination of medical conditions, and therefore appear to have a lower capacity for engagement in PA. With our study design, it was not possible to clarify the extent to which participants chose the response option “potentially” because they felt that no clear answer could be given. A definite recommendation might depend on the individual clinical case. For example, we only asked about osteolysis as a potential contraindication, although a precise recommendation can only be made after a stability or fracture risk assessment.

However, as the first cross-sectional study investigating HCPs’ understanding of contraindications to advise PA in the setting of HSCT, the results of our study provide valuable insight in specific safety issues which can hinder HCPs to recommend PA. By making use of various recruiting strategies, we aimed at obtaining a random and highly representative sample. Our questionnaire was inspired by and is partly consistent with the questionnaire used in the MOMENTUM Project, a cross-sectional survey developed elaborately using a sound theoretical approach and various qualitative and quantitative pretests [45].

Familiarity and practical experience of physicians with medical conditions such as cardiac insufficiency, osteolysis, pulmonary disease, and hemoglobin < 8 g/dl might lead to being more permissive to PA. Therefore, it should be strongly recommended that physical therapists and nursing staff consult treating physicians before delivering PA interventions and/or PA counseling, and discuss any additional signs to monitor during therapy sessions. Accordingly, there is a clear need for a good multidisciplinary cooperation between all HCPs treating and caring for HSCT patients. Furthermore, physical therapists have to use their clinical judgment and expertise to modify components of programs to fit patient needs. This means that PA during and post HSCT has to focus on individual needs in which laboratory values (such as platelet counts and hemoglobin) are utilized to make program adjustments. Education possibilities and evidence-based courses to build knowledge regarding safety concerns should be standard practice in oncology care, as HCPs’ concerns will directly influence the patients’ PA behavior [16]. Instead of considering certain medical conditions as contraindications or being reluctant to advise or refer HSCT patients to PA, training intensity needs to be adjusted: PA should be less intense, increased slowly, and overtraining must be avoided [24].

## Conclusion

As HSCT patients often present with complex health issues, certain medical conditions and safety concerns might prevent HCPs to promote health enhancing PA before, during, and after stem cell transplantation. Although “acute infection” was the only condition which was predominantly considered as contraindication by all HCPs, the large number of potentially-answers might reflect caution or uncertainty and might consequently influence HCPs’ PA counseling behavior. A discrepancy in the understanding of certain medical conditions between physicians, nursing staff, and physical therapists becomes apparent, whereas physicians had the least concerns regarding PA. Education possibilities and evidence-based courses to build knowledge especially regarding often-discussed safety concerns should be the standard practice in the setting of HSCT. Furthermore, there is a clear need for a good multidisciplinary cooperation between all HCPs in order to support patients to confidently engage in PA. The present findings underline the need for further clinical and empirical research.

## Data Availability

The authors declare that they have full control of all primary data and that they agree to allow the journal to review their data.

## References

[1] Niederwieser D, Baldomero H, Szer J (2017). Hematopoietic stem cell transplantation activity worldwide in 2012 and a SWOT analysis of the Worldwide Network for Blood and Marrow Transplantation Group (WBMT) including the global survey. Bone Marrow Transplant.

[2] Van HIEPM, Timmerman H, Potting CM (2013). Physical exercise for patients undergoing hematopoietic stem cell transplantation: systematic review and meta-analyses of randomized controlled trials. Phys Ther.

[3] Prins MC, van Hinte G, Koenders N (2021). The effect of exercise and nutrition interventions on physical functioning in patients undergoing haematopoietic stem cell transplantation: a systematic review and meta-analysis. Support Care Cancer.

[4] Schmitz KH, Campbell AM, Stuiver MM (2019). Exercise is medicine in oncology: engaging clinicians to help patients move through cancer. CA Cancer J Clin.

[5] Morishita S, Tsubaki A, Hotta K (2019). The benefit of exercise in patients who undergo allogeneic hematopoietic stem cell transplantation. J Int Soc Phys Rehabil Med.

[6] Wiskemann J, Kleindienst N, Kuehl R (2015). Effects of physical exercise on survival after allogeneic stem cell transplantation. Int J Cancer.

[7] Knips L, Bergenthal N, Streckmann F (2019). Aerobic physical exercise for adult patients with haematological malignancies. Cochrane Database Syst Rev.

[8] Park JH, Lee J, Oh M (2015). The effect of oncologists’ exercise recommendations on the level of exercise and quality of life in survivors of breast and colorectal cancer: a randomized controlled trial. Cancer.

[9] Meyer-Schwickerath C, Morawietz C, Baumann FT (2021). Efficacy of face-to-face behavior change counseling interventions on physical activity behavior in cancer survivors–a systematic review and meta-analysis. Disabil Rehabil.

[10] Hardcastle SJ, Cohen PA (2017). Effective physical activity promotion to survivors of cancer is likely to be home based and to require oncologist participation. J Clin Oncol.

[11] Kenzik K, Pisu M, Fouad MN, Martin MY (2016). Are long-term cancer survivors and physicians discussing health promotion and healthy behaviors?. J Cancer Surviv.

[12] Hausmann A, Gabrian M, Ungar N (2018). What hinders healthcare professionals in promoting physical activity towards cancer patients? The influencing role of healthcare professionals’ concerns, perceived patient characteristics and perceived structural factors. Eur J Cancer Care (Engl).

[13] Keogh JWL, Olsen A, Climstein M (2017). Benefits and barriers of cancer practitioners discussing physical activity with their cancer patients. J Cancer Educ.

[14] Newton RU, Hart NH, Clay T (2020). Keeping patients with cancer exercising in the age of COVID-19. JCO Oncol Pr.

[15] Hayes SC, Newton RU, Spence RR, Galvão DA (2019). The Exercise and Sports Science Australia position statement: exercise medicine in cancer management. J Sci Med Sport.

[16] Schmitz KH, Courneya KS, Matthews C (2010). American College of Sports Medicine roundtable on exercise guidelines for cancer survivors. Med Sci Sport Exerc.

[17] Hausmann A, Ungar N, Tsiouris A (2021). Physical activity counseling to cancer patients: how are patients addressed and who benefits most?. Patient Educ Couns.

[18] Steindorf K, Depenbusch J, Haussmann A (2020). Change patterns and determinants of physical activity differ between breast, prostate, and colorectal cancer patients. Support Care Cancer.

[19] Depenbusch J, Wiskemann J, Haussmann A (2021). Impact and determinants of structural barriers on physical activity in people with cancer. Int J Behav Med.

[20] Tsiouris A, Ungar N, Haussmann A (2018). Health care professionals’ perception of contraindications for physical activity during cancer treatment. Front Oncol.

[21] Saad A, De LM, Anand S (2020). Hematopoietic cell transplantation, version 2. 2020. JNCCN.

[22] Cohen J (1988). Statistical power analysis for the behavioral sciences.

[23] Campbell KL, Winters-Stone KM, Wiskemann J (2019). Exercise guidelines for cancer survivors: consensus statement from International Multidisciplinary Roundtable. Med Sci Sports Exerc.

[24] Maltser S, Cristian A, Silver JK (2018). A focused review of safety considerations in cancer rehabilitation. PM.

[25] Wolin KY, Schwartz AL, Matthews CE (2012). Implementing the exercise guidelines for cancer survivors. J Support Oncol.

[26] Jones LW, Eves ND, Peppercorn J (2010). Pre-exercise screening and prescription guidelines for cancer patients. Lancet Oncol.

[27] Stout NL, Brown JC, Schwartz AL (2021). An exercise oncology clinical pathway: screening and referral for personalized interventions. Cancer.

[28] Friman G, Wesslen L (2000). Infections and exercise in high-performance athletes. Immunol Cell Biol.

[29] Dick NA, Diehl JJ (2014). Febrile illness in the athlete. Sports Health.

[30] Hedin G, Friman G (1982). Orthostatic reactions and blood volumes after moderate physical activation during acute febrile infections. Int Rehabil Med.

[31] Jarden M, Baadsgaard MT, Hovgaard DJ (2009). A randomized trial on the effect of a multimodal intervention on physical capacity, functional performance and quality of life in adult patients undergoing allogeneic SCT. Bone Marrow Transplant.

[32] Winningham ML, MacVicar MG, Burke CA (1986). Exercise for cancer patients: guidelines and precautions. Phys Sportsmed.

[33] Tosetto A, Balduini CL, Cattaneo M (2009). Management of bleeding and of invasive procedures in patients with platelet disorders and/or thrombocytopenia: guidelines of the Italian Society for Haemostasis and Thrombosis (SISET). Thromb Res.

[34] Elter T, Stipanov M, Heuser E (2009). Is physical exercise possible in patients with critical cytopenia undergoing intensive chemotherapy for acute leukaemia or aggressive lymphoma?. Int J Hematol.

[35] Grencheski EA, Kochi MN, Politi FVA (2021). Bleeding frequency during physiotherapy in thrombocytopenic patients undergoing hematopoietic stem cell transplantation. PLoS ONE.

[36] Fu JB, Tennison JM, Rutzen-lopez IM (2018). Bleeding frequency and characteristics among hematologic malignancy inpatient rehabilitation patients with severe thrombocytopenia. Support Care Cancer.

[37] Morishita S, Kaida K, Setogawa K (2013). Safety and feasibility of physical therapy in cytopenic patients during allogeneic haematopoietic stem cell transplantation. Eur J Cancer Care (Engl).

[38] Mohammed J, Aljurf M, Althumayri A (2019). Physical therapy pathway and protocol for patients undergoing hematopoietic stem cell transplantation: recommendations from The Eastern Mediterranean Blood and Marrow Transplantation (EMBMT) group. Hematol Oncol Stem Cell Ther.

[39] Campbell KL, Cormie P, Weller S, Alibhai SMH (2022). Exercise recommendation for people with bone metastases: expert exercise recommendation for people with bone metastases: expert consensus for health care providers and exercise professionals. JCO Oncol Pract.

[40] Weller S, Hart NH, Bolam KA (2021). Critical reviews in oncology / hematology exercise for individuals with bone metastases: a systematic review. Crit Rev Oncol / Hematol.

[41] Karvinen KH, DuBose KD, Carney B, Allison RR (2010). Promotion of physical activity among oncologists in the United States. J Support Oncol.

[42] Park J-H, Oh M, Yoon YJ (2015). Characteristics of attitude and recommendation of oncologists toward exercise in South Korea: a cross sectional survey study. BMC Cancer.

[43] Karvinen KH, McGourty S, Parent T, Walker PR (2012). Physical activity promotion among oncology nurses. Cancer Nurs.

[44] Jones LW, Courneya KS, Peddle C, Mackey JR (2005). Oncologists’ opinions towards recommending exercise to patients with cancer: a Canadian national survey. Support Care Cancer.

[45] Ungar N, Tsiouris A, Haussmann A (2019). To rest or not to rest—health care professionals’ attitude toward recommending physical activity to their cancer patients. Psychooncology.

